# Theory of Insanity
*Remarks on Insanity, its Nature and Treatment. By Henry Monro, M.D. In 2 parts. Part I. 1850.


**Published:** 1851-01-01

**Authors:** 


### Art. VII.—THEORY OF INSANITY.*

The name of Monro has for so long a period been associated with the subject of insanity and with Bethlehem Hospital,—one of the oldest lunatic asylums in Europe,—that any treatise on affections of the mind proceeding from the pen of a member of that respectable and distinguished family will and ought to be received with unusual interest by the profession. Sir Walter Scott observed, "that a successful author has not a more dangerous rival than himself; his successive productions will always be tried by the standard of his *chef d'œuvre,*" and hence, he remarks, "it is sometimes more easy to *acquire* than to *sustain* a reputation." We by no means assert this to be the position of Dr. Henry Monro, in reference to the well-earned status of his ancestry. Let him be of good cheer; he has, we are informed, prepared himself to deal with the subject by study and steady practical observation. It may be true that he has not struck positively into any new path, or turned aside the veil which conceals from our finite understandings much that is mysterious with regard to the mind, in health or disease; or revealed to us any previously undiscovered star, to guide us safely through the intricate labyrinth which he attempts to explore; but he has brought with him an obvious spirit of philosophic inquiry and independence, and has developed many interesting and peculiar, if

* Remarks on Insanity, its Nature and Treatment. By Henry Monro, M.D. In 2 parts. Part I. 1850.

not novel views, upon a variety of points which, it must be confessed, are (and we are afraid ever will remain) involved in deep and impenetrable obscurity. With much that he has written it affords us pleasure to say we cordially agree, but with much we confess we differ; but our very difference is, we hope, tempered with a feeling of respect for the author. Dr. Monro's object is to establish that insanity is a disease caused by "*loss of nervous tone*," and that this condition arises from depressed vitality. The theory is thus propounded by its author:—

"Insanity is a disease of loss of nervous tone; that this loss of nervous tone is caused by a premature and abnormal exhaustibility of the vital powers of the sensorium; that this infirmity is essentially a local one, though torpor of the general, physical, and vital powers assists it; and its origin is to be esteemed constitutional, congenital, and frequently hereditary."

Again, the author states (page 12):

"The theory of the pathology of insanity which I wish to put forward in this treatise is, that it is an affection consequent on depressed vitality, which depressed vitality is wont to manifest itself with peculiar and specific force in the cerebral masses, owing to a congenital and frequently an hereditary tendency, as the brain must succumb when oppressed by any exciting cause."

In support of these views he refers, first, to the analogy existing between the symptoms of insanity, and certain mental conditions which are acknowledged to arise from deficiency of nervous power, obviously consequent on depressed vitality; second, to the general state of the physical condition of the insane; and, thirdly, he refers to the benefit he has found arise from the treatment which has been based upon this hypothesis. Thus far (assuming his positions to be tenable) the views of the author would appear to be substantiated, for in a numerous class of insane cases, it is generally admitted that there exists a marked want of tone and energy in the nervous system, accompanied by an obvious depression of vital energy. There can, indeed, be no doubt that insanity often arises, as Dr. Monro suggests, from *exhaustion and loss of nervous tone*; that in these cases the manifestation of strength, so frequently exhibited, is purely *fictitious*; that in the words of that acute physician, Dr. Seymour, we have, in the majority of cases, "*excitement without power*;" but this theory will not account for the insanity which results, as it occasionally does, from the opposite condition of the system, viz. that associated with an acute or sub-acute affection of the brain and its investments, requiring for its treatment measures somewhat active in their character. Hence, on the very threshold of the inquiry we meet with this difficulty, that in many cases, particularly in the incipient stage of insanity, there is an *obvious and positive augmentation of nervous energy, and an exaltation of the intellectual faculties*. How is this to be explained? The author meets the objection by arguing, that in these cases the depressed state of vitality—this "deficiency of nervous tone"—gives rise to an "irritable excess of action," which accounts for all the phenomena of excitation. He acknowledges that, "as regards most of the lower but more elementary qualities of the mind, they exist very often to an intenser degree in the insane than they do in the sane;" he admits that "the most elementary of all the phenomena of mind, namely, the consciousness of the conception of images by the mind, is morbidly active, either in the way of rapidity of succession of ideas, or indelible impression of single ideas;" he concedes, moreover, that "the instinctive impulses, which are the highest qualities of mind in many of the lower tribes of animals, are very often so excessive in insanity, as to become, in some cases, the most remarkable characteristics of the disease"—nay, that "even the higher intellectual faculties, though for the most part more or less suspended by insanity, are in some remarkable cases morbidly excessive;" and yet all this exaltation he ascribes to some "coincident excess," arising from irritation consequent upon impaired nervous energy and depressed vitality. Look at the insane patient with his flushed face, injected conjunctivæ, and incoherent raving—his extreme excitement, and manifestation of physical strength; listen to the rapid flow of unconnected ideas, which seem to flash like lightning through his mind; feel the accelerated pulse, the heated scalp, and observe the abnormal rapidity of the circulation through the system; and then ask, What evidence is there in *this state* (often connected with uncomplicated insanity) of "*loss of nervous tone*," or "*diminished vitality?*" True it is, that in a very large class of cases there is an *asthenic* condition, such as the author has clearly and well described; but we must be cautious of hasty generalizations; in steering clear of Charybdis, we must not wreck ourselves upon Scylla; in avoiding one theory, we must not peril our whole induction upon another. The doctrine of depressed vitality and impaired nervous energy can by no means account for an abnormal accumulation of nervous energy; for those contrary and antagonistic conditions, which can never be brought, by any process of reasoning, into the relation of cause and effect. "The violent efforts of thought and action manifested by the insane are nothing more nor less," observes the author, "than attempts on the part of nature to free itself of a morbid excess of nervous energy," (page 17.) This we grant, but *unde derivatur?* The word "irritability" has, since the days of Haller, been used by physiologists in a very vague and ambiguous manner. *Ubi irritatio, ibi fluxus;* but this state of irritation is only an antecedent link in the chain. Nay, there is no disease, whether of the vascular or nervous system, that is not, at all events in its earlier stages, accompanied by abnormal irritability; and this very irritability, we submit, augments rather than diminishes nervous energy; and when the sensorial functions are hereby stimulated into a state of abnormal activity, surely the excess of action is to be ascribed to the excess rather than the diminution of nervous energy. That cerebral irritability produces a consequent train of disturbed mental phenomena there can be no doubt, but that it is to be laid down as a law, that insanity is *always* a consequence of loss of nervous tone, we are not prepared to admit.

The author dwells at some length upon the corporeal nature of insanity:

"I believe," says he, "that insanity is simply a disease of the nervous instruments of the mind." (This we apprehend to be the opinion generally entertained.) "The person," he observes, "who repudiates the idea of the physical nature of insanity in its various stages of delirium and imbecility, should also repudiate the doctrine of the physical nature of the delirium of fever, and the imbecility of old age; for though insanity is a specific disease in some of its aspects, yet in most points, at a certain stage, it is so allied to the conditions of mental phenomena in fever, and at a certain other stage to those of old age, that it is nearly impossible to make any psychological distinction between them. Neither do I see why he should not repudiate also the idea, that the condition of a born idiot is dependent on bodily defect; and I do not see how he can help coming to the further conclusion, that the abstract mind (a being not subject to decay) of a cretin is a different sort of mind from that of other people."—(p. 6.)

All this is sufficiently apposite; but in endeavouring to establish the independence of the mind, the author has recourse to an illustration which involves the whole question in inextricable obscurity. He assumes, as an established point, that motion is not a condition of matter; but an entirely separate and independent principle.

"Let us take another act," he observes, "performed by means of the body, which is also the manifestation of *a great principle, really external to, and independent of, all animal bodies.* Who would pretend to say, that motion is identical with those changes in the muscular and nervous parts of animal mechanism, by the conjoint operation of whose functions motion is effected, and that motion is only commensurate with nervous stimulus and muscular contractility? No one could; because we see motion exists commensurately with matter, and it pervades all that sphere to which its operations could belong, or pretend; we can state this as a fact, through the instrumentality of our senses; and *why should we doubt that, as motion is independent of the body it acts through,* as motion embraces a sphere of action equal to its properties and pretensions, so mind is independent of the body it acts through, and is to be deemed commensurate with the extent of its properties and pretensions, which (in the human mind distinctively,) are to aspire after eternity,—

to possess the knowledge of moral good and moral evil, and to desire the perfection of moral good—faculties, in short, which cannot have their full end and object in this life, and must have them in a sphere suited to their full development."—(p. 7.)

The ancient philosophers, it will be remembered, puzzled themselves sorely to discover the origin and nature of motion, which many of them conceived to be identical with life; but we cannot understand what analogy—even adduced by way of illustration—it bears to the mind, or upon what theory of mechanical philosophy it is to be regarded as a principle independent of matter. This hypothesis carries us to the very brink of German pantheism, as recently propounded by Stallo, who lays it down as an axiom, that "matter exists not in itself, but by virtue of, and in reference to, its inner vitality;" and that "the true centre of the gravity of matter is its inner life." But we digress: allowing insanity to be merely a disease of the brain, which becomes an imperfect instrument of the mind, we can by no means understand the localization which the author would give to the different intellectual faculties. "In the divisions I am about to make," he observes, "of the seats of the various mental faculties, I hope that I may escape the charges brought against phrenological distinctions generally; for be it observed, that though I recognise (as I believe the best physiologists of the present day will agree with me in doing) the probable distinctiveness of locality for the operation of broadly-distinct faculties, I by no means attempt to localize their sphere of action" (p. 12); yet in the next page the author speaks of "the seats of the more elementary faculties, such as the conception of ideas," &c., and "the seats of the higher faculties, such as reason and will," &c., as if he fairly adopted some system or other of organology closely resembling, at all events, phrenology. Probably, in the second part of his "Remarks," the author will, on this interesting subject, explain his views more fully, and point out these "promised divisions," or seats of those "broadly-distinct faculties," which he recognises as entering into the elementary constitution of the human mind.

In the second chapter of his "Remarks," Dr. H. Monro enters upon the consideration of various modifications, or forms as he terms them, of "partial intellectual insanity;" and here, as well as in his "Introduction," he appears to involve himself in some difficulty respecting what he considers to be complete insanity, which implicates very seriously the question of morals. "The complete maniac," he tells us, "lives in a waking dream,—he raves without power to stop himself,—without the power to appreciate the necessity of stopping himself; he is completely the victim, not in the least the master, of the strongest impressions uppermost in his fancy." Here there can be no doubt all moral responsibility is at an end; not so, however, he argues, where insanity is "not complete;" in such cases "morals have some place." This brings us to the question, what the author means by cases of incomplete mania? He asked a young gentleman why he did not restrain himself from making absurd grimaces and gestures; and was answered "with great ingenuousness, that he (the patient) felt the long continuance of such restraint intolerable, though he saw the propriety of it." A young lady, after her recovery, told him that "she felt throughout her illness, that she could have restrained her extreme acts of violence if she had chosen; but that at certain times such an attempt seemed intolerable, and so she gave way to her propensities when stimulated by heat of head," &c. A medical man, he further relates, "used to request that he might be kept under restraint, to prevent him from smashing every thing about him." In all these cases the author argues, "that the insanity was not complete, because the judgment and the power of self-restraint were not absolutely gone, though the attempt to exercise them was an intolerable labour." (*Introduction*, v.) In all these cases, however, the contrary would seem to be the fact; there was in each a strong desire to resist the insane impulse, but an utter incapacity to do so. Theirs was not an incomplete, but a complete development of impulsive insanity; and to hold either morally responsible for actions they could not control, would be the highest degree of inhumanity. The line of demarcation drawn by Dr. Monro seems to be as follows: the *complete* maniac is an irresponsible being; but persons who are *partially*—as he designates it—insane, may be fit subjects for moral discipline. The difficulty of pointing out the exact point at which the power of self-control ceases, we have frequently dwelt upon; in the incipient stages of insanity, we have frequently urged the necessity of such patients summoning up all their powers of volition to resist the morbid impulses of which they are conscious; but all their energies become unavailing as the disease, unhappily, progresses. But the author does not use the word "complete," or "partial," in the sense in which they are usually applied. When monomania is spoken of as a form of "partial insanity," we simply imply that the individual labours under a specific delusion, apart from which his other mental faculties are (to all appearance) unaffected. The disease is partial, as only affecting a particular series or train of ideas; but in respect to the monomania itself, it cannot be called partial, for the delusion is *perfect*, and this form of insanity, therefore, is thoroughly confirmed. When we speak of partial intellectual insanity, we only mean that certain of the mental faculties are deranged, while others remain intact. The man who believes himself to be Julius Cæsar may entertain correct notions enough on other subjects, upon which he will reason with intelligence; but his insanity is not the less complete upon the subject of his derangement. The word partial, therefore, as applied to this form of insanity is, strictly speaking, inaccurate. A man conceives himself to be our Saviour. His delusion is complete; there is nothing partial whatever about it. The account which the author gives of the consciousness which some patients have of impending insanity, and the terrible struggle which ensues in the endeavour to resist its invasion is exceedingly forcible. It is a very painful but correct picture. He next directs his attention to a class of persons in whom the disease manifests itself by a distressing state of restlessness, and an undefined impression that something dreadful is about to occur. "They feel," he remarks, "deep anxiety and torturing irritation of mind, and yet they know not what it is all about: fancy stimulates, reason does not respond; and though the will to alter and allay their thoughts is striven after, it is not fully grasped, or effectually obtained. This condition resembles a person in the night-mare, who strives to open his eyes, who knows it is all a dream, and yet is so spell-bound that he cannot release himself from it." (p. 26.) It is well observed by the author, that persons who give way to, and indulge in, excessive impressions which exclude other considerations, are indulging a rudimentary form of mental disease; for by yielding themselves up entirely to their morbid impressions, they eventually lose the power of exercising their reasoning faculties. The following observations on this subject are exceedingly just:

"Persons of the irritable diathesis are peculiarly subject to degrees of this condition of mind; and considering the results which such an inclination may lead to, we must see how very important it is to resist every such tendency with every effort of our will, as long as power over that will is granted us. Moreover, we must ever remember, that that which, up to a certain point, may be beautiful and interesting, (I mean a habit of strong and overpowering emotions of the mind,) may, by a little farther excess, become a frightful disease; and that though the flow of imagery may be charming to the mind inclined to such phenomena (as there is little or no effort required for its exercise in those who are thus naturally disposed), yet the real source of the charm consists in the conviction, that we can stop it at will, and relinquish its wandering guidance, when the real emergencies of our condition require it: the charm is generally all gone, both to the sufferer and the beholder, when this power is lost, and the dreamer becomes the victim, and not the master of his visions. We can weep with pleasure when we contemplate some of the touching images raised by poor Ophelia's wanderings: this pleasure arises from a momentary sympathy with such a state of weakness and dependence; but the pleasure of the sympathy would indeed vanish, should a doubt arise as to whether our minds had that power of elasticity which enables us to rise again above such companionship; and, were our minds compelled to wander with Ophelia's all our lives, we should sigh indeed (had we the power of regret left to us) for that state of vigour of mind necessary for any enjoyment of the things of life. Those who wish to be convinced of these things need only witness madness in reality, and compare it with madness in poetry; or rather, I would say, witness the feelings which one mad person entertains towards another, and compare it with the feelings that a sane person can afford to have towards one afflicted in this way."—(pp. 31-32.)

We do not concur altogether in Dr. H. Monro's observation, that in moral insanity—in which the moral sense is unaccountably perverted, while the judgment remains pretty clear—there is generally a greater amount of intellectual deficiency than is generally acknowledged. Our own experience has convinced us that in many such cases the intellectual faculties are unusually clear, active, and suggestive. The author, however, abstains from dwelling upon this form of the disease, under the conviction that it is "difficult and replete with danger, both socially as well as religiously, to decide where actual physical disease steps in, of such an amount as to incapacitate the mind from its proper action." However difficult, this we conceive to be one of the most important investigations upon which the medico-psychologist can enter. As a point of medical jurisprudence deeply affecting the welfare of society, it is one that has the strongest claim upon our attention.

The author next proceeds to examine the pathology of various mental phenomena which resemble, or may be compared with insanity, and which seem to support his theory of all such conditions arising from loss of nervous energy, or an exhausted condition of the vital powers of the sensorium. These abnormal states he considers under the heads of sleep and dreaming, somnambulism, waking trance, voluntary abstraction, transporting passion, the mental state in infancy and old age, and imperfections of the mind arising from intoxication, fever, and various internal and external causes. Dr. Abercrombie, in his "Inquiries concerning the Intellectual Powers," observes, that "insanity and dreaming, considered as mental powers, have a remarkable affinity to each other. The great difference between them," he observes, "is, that in insanity the erroneous impression being permanent affects the conduct, whereas in dreaming no influence on the conduct is produced, because the vision is dissipated upon waking." (*Intellectual Powers*, p. 269.) Dr. Macnish, in his "Philosophy of Sleep," observes, "that Dr. Abercrombie's definition is nearly, but not wholly correct, for in somnambulism and sleep-talking, the conduct is influenced by the prevailing dream." "Dr. Rush," he adds, "has remarked with great shrewdness, that a dream may be considered as a transient paroxysm of delirium; and delirium, as a permanent dream. (*Philosophy of Sleep*, p. 44.)

The analogy which Dr. H. Monro has drawn between the mental state in sleep and dreams, and the phenomena of insanity, merits attention, and will be read with interest:

"The mental phenomena of sleep, when profound, are not remembered, if any exist: we must therefore take those of less profound or dreaming sleep. Here the condition of the mind is very like that of *intense* insanity—namely, a very vivid impression of simple images passing before the mind,—an inability to compare these images with the things of the external world,—an inability to judge of the relation one image bears to another,—and, above all, an inability to control the train of these images by an act of will, either as regards their origin, their course, or their interruption. The most striking distinctions between the phenomena of dreams and those of intense insanity are— 1st. That the external world is never perhaps so entirely shut out in insanity as it is in dreams, the special senses seldom or never being so much suspended; 2ndly. The power of voluntary motion is lost generally in sleep, but it exists in insanity; 3dly. The dreaming state is temporary, and able to be dispelled, while insanity is more or less permanent. There is, however, a less profound sleep even than that of the ordinary dreaming state, which generally occurs when a person is very near the waking state, though some excitable temperaments are subject to its phenomena more or less at all times. In this condition the external world is not wholly shut off from the dreamer; for he is conscious of sounds, &c., though he misinterprets them; he is able also to use his organs of motion, as is manifested in talking in sleep and throwing his limbs about: this, however, approaches the condition of somnambulism. In the state of very light sleep the reasoning faculties are often as intense as in the waking state, though moral liberty is not even yet achieved; and thus the succession of ideas is not directed by the will, but by other influences, such as those impressions most deeply engraven at the time on the memory, or those sensations most strong on the field of consciousness. It may be said of this condition what Locke said of insanity—they argue rightly, but on wrong premises."—pp. 36-7.

We are somewhat surprised that the author, in accounting for the phenomenon of sleep, goes beyond the limits of his favourite theory of exhausted nervous energy, and states that he believes this depressed condition arises from "deficiently vitalized blood—or in other words, that through the loss of vitality in the assimilating and purifying processes of the blood, carbon and other deleterious ingredients (?) are accumulated and not given off, in consequence of which the blood becomes too venous, and not sufficiently arterial, and this, as is well known, will cause stupefaction of the nervous power." This "carbon" theory of the cause of sleep, which was many years ago put forward with considerable ingenuity, has always appeared to us very objectionable. Upon this hypothesis, "Nature's sweet restorer, balmy sleep," instead of being a natural and healthful state of repose, would be an abnormal condition, a precursory stage of asphyxia. Physiologists have clearly proved that an excess of carbon, without any "other deleterious ingredients," in the venous system, acts as a specific poison upon the nervous tissue. Besides, as this carbonic sedative must go on increasing, how does the sleeper after a certain period awake? "*Sublata causa tollitur effectus;* but the cause is here not removed; nay, it may be supposed to be aggravated, for the circulation and respiration during sleep are retarded. It appears to us, that sensorial exhaustion, consequent upon diurnal stimulation, is quite sufficient to account for all the physical characteristics, at least, of sleep, however difficult it may be to explain its psychological phenomena. "Our life is twofold—sleep hath its own world."

The condition of the mind in infancy, Dr. H. Monro has dwelt upon at some length; but we must confess that we do not perceive any analogy between the mind of an infant and the mind of an insane person. It does not follow, that because a child cannot reason, inasmuch as its psychological powers are not yet developed, that therefore its mind is in a condition analogous to that of a mind in which reason has been dethroned; as well might it be argued that an infant, because it totters about a room before it has acquired command over its limbs, is in a condition analogous to that of a paralytic person, who has lost the power of voluntary motion. Here, however, we shall allow the author to speak for himself:—

"The condition of the mental phenomena in early years is in some point similar to the condition of the insane mind, though the mode in which this condition is arrived at in these two cases is very different; the one not having arrived at the maturity of mental manifestation, while the other has lost a maturity which he once possessed. The mind of youth is peculiarly prone to a most vivid conception of simple ideas, while it has not much power of connecting and ordering those ideas. The fervid impression of simple suggestions in early years is well known to be such as never exists again after the full powers of the mind are developed; their poignancy and keenness, indeed, are never afterwards forgotten, if once stored up in a retentive mind; and the recollection of these feelings, as well as the ability to realise again these feelings through an effort of memory, forms one of the most innocent as well as glowing sources of happiness which this world can afford. This accounts for the very vivid feelings little children have about colour, sound, and impressions on common sensations: any one who remembers well his early impressions on these subjects, and compares them with the impressions raised by the same objects in after years, will be struck with the constrast. The blue of the sea or the sky, the variegated blossoms of spring, the red and purple of sunsets, are fixed with a distinctness of pleasurable sensation not able to be realised in after years, and which, if not remembered from the days of youth, can never be appreciated. The sweet scent of a hay-field in a summer's evening, the song of birds at the close of day, the pleasurable and painful sensations raised by warmth and cold, the terror raised by some impressions on the mind, the dim recollection of scenes of very early life so pregnant with intense delight or intense anguish, all speak with one voice to the truth of what I assert. And this is the reason why reflective people dwell with such peculiar interest on this undeveloped period of existence: to them the mental phenomena of this period are like highly-finished pictures, while the after-impressions of a similar nature resemble rather cold outlines; and we may say that the sensations conveyed by dreams are more like these early impressions than anything realised in the waking state of manhood. Children, however, have not the power of reasoning, and comparing, and directing their acts of volition, to anything like the same extent that the full-developed mind of man has; and in these respects the mind of a child is similar to that of the insane."—pp. 52-53.

We have no hesitation in saying that insanity occurs in early life more frequently than is commonly supposed, or is even suspected; but this analogy is drawn under the supposition of the infant mind being in a state of "supreme health." Cases of insanity in extremely advanced life are of constant occurrence; and as an illustration of that fact, and at the same time as a fair specimen of the author's style, we cannot do better than conclude our notice with his description of the mental state in old age.

"The phenomena of the mind in the other extreme of life—namely, senile imbecility,—as well as the cause of these phenomena, so much resemble those of many forms of insanity, that frequently it is difficult to draw the line between what is the result of healthy decay and what is to be considered disease. These phenomena, however, resemble those of the *second* stage of insanity rather than the first—namely, that state where all the mental faculties are becoming gradually suspended, and when no excess of any particular faculties exists: the phenomena of simple suggestion succumb equally with those of relative suggestion; the simple images of the mind gradually fade, equally with the powers of analysis and synthesis; the special senses become blunted coincidently with the more abstract efforts of the conscious being. To use the words of metaphor, in old age the stream does not, as in insanity, attempt to compensate for its deficient volume by its fury; for the channel is obliterated with the dissolution of the stream. Or, to carry out this metaphor in detail, we may say that the phenomena of old age resemble that quiet dissolution which occurs when a mighty river is approaching the close of its proper destiny, and is about to retire for ever into that ocean whither its course has ever tended; its waters, as they gradually disappear, leave no marks of ruin behind, but rather a kindly soil, as a memorial of that which is passed; so that, when the pleasant hours of its vigour have passed away, and its power to soothe the traveller by the murmuring sounds of its waters and the refreshing sight of its expanse have departed, its channel and its tide can be no more traced: whereas the phenomena of insanity are like the disturbed and premature dissolution of that river, whose waters fail before its work is done, and whose channel remains entire while its tide diminishes. Fury must now do what vigour has ceased to fulfil, and the cataract try to compensate for the loss of the tide of many waters: the stream, as it seeks the ocean, continues for a space to remind the passenger of the loss that nature's harmony has sustained, and to fill his mind with the impressions of storm and wreck instead of serenity and rest."—p. 55.

